# Cardiovascular risk factors and lifestyle behaviours in relation to longevity: a Mendelian randomization study

**DOI:** 10.1111/joim.13196

**Published:** 2020-11-13

**Authors:** S. van Oort, J. W. J. Beulens, A. J. van Ballegooijen, S. Burgess, S. C. Larsson

**Affiliations:** ^1^ Department of Surgical Sciences Uppsala University Uppsala Sweden; ^2^ Department of Epidemiology and Data Science Amsterdam Public Health Research Institute and Amsterdam Cardiovascular Sciences Research Institute Amsterdam University Medical Centers Vrije Universiteit Amsterdam Amsterdam the Netherlands; ^3^ Julius Center for Health Sciences and Primary Care University Medical Center Utrecht Utrecht the Netherlands; ^4^ Department of Nephrology Amsterdam University Medical Centers Vrije Universiteit Amsterdam the Netherlands; ^5^ MRC Biostatistics Unit University of Cambridge Cambridge UK; ^6^ Department of Public Health and Primary Care University of Cambridge Cambridge UK; ^7^ Unit of Cardiovascular and Nutritional Epidemiology Institute of Environmental Medicine Karolinska Institutet Stockholm Sweden

**Keywords:** cardiovascular risk factors, instrumental variable analysis, lifestyle, longevity, Mendelian randomization

## Abstract

**Background:**

The American Heart Association introduced the *Life's Simple 7* initiative to improve cardiovascular health by modifying cardiovascular risk factors and lifestyle behaviours. It is unclear whether these risk factors are causally associated with longevity.

**Objectives:**

This study aimed to investigate causal associations of *Life's Simple 7* modifiable risk factors, as well as sleep and education, with longevity using the two‐sample Mendelian randomization design.

**Methods:**

Instrumental variables for the modifiable risk factors were obtained from large‐scale genome‐wide association studies. Data on longevity beyond the 90^th^ survival percentile were extracted from a genome‐wide association meta‐analysis with 11,262 cases and 25,483 controls whose age at death or last contact was ≤ the 60^th^ survival percentile.

**Results:**

Risk factors associated with a lower odds of longevity included the following: genetic liability to type 2 diabetes (OR 0.88; 95% CI: 0.84;0.92), genetically predicted systolic and diastolic blood pressure (per 1‐mmHg increase: 0.96; 0.94;0.97 and 0.95; 0.93;0.97), body mass index (per 1‐SD increase: 0.80; 0.74;0.86), low‐density lipoprotein cholesterol (per 1‐SD increase: 0.75; 0.65;0.86) and smoking initiation (0.75; 0.66;0.85). Genetically increased high‐density lipoprotein cholesterol (per 1‐SD increase: 1.23; 1.08;1.41) and educational level (per 1‐SD increase: 1.64; 1.45;1.86) were associated with a higher odds of longevity. Fasting glucose and other lifestyle factors were not significantly associated with longevity.

**Conclusion:**

Most of the *Life's Simple 7* modifiable risk factors are causally related to longevity. Prevention strategies should focus on modifying these risk factors and reducing education inequalities to improve cardiovascular health and longevity.

## Graphical abstract

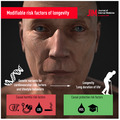

**Abbreviations**
BMI: Body mass index
CVD: Cardiovascular disease
DBP: Diastolic blood pressure
GWAS: Genome‐wide association study
HDL: High‐density lipoprotein
IVW: Inverse variance‐weighted
LDL: Low‐density lipoprotein
MR: Mendelian randomization
MR‐PRESSO: Mendelian Randomization Pleiotropy RESidual Sum and Outlier
MVPA: Moderate‐to‐vigorous physical activity
SBP: Systolic blood pressure
SNP: Single‐nucleotide polymorphism

## Introduction

Over the past decades, life expectancy has increased enormously [1]. Still, it is largely unclear why certain individuals survive to extreme ages and become the longest‐lived of their generation, whilst others die earlier. Longevity tends to cluster within families, probably as a result of shared genetic and environmental factors [2]. The variation in life span is estimated to be heritable for ~25% [1], which leaves 75% to be influenced by environmental factors. Better insight into the association between potentially modifiable risk factors and longevity can inform strategies for a long and healthy life.

In 2010, the American Heart Association introduced the *Life’s Simple 7* initiative [3]. The goal was to optimize cardiovascular health by focusing on seven modifiable risk factors, including three cardiovascular risk factors (glucose, blood pressure and cholesterol) and four lifestyle behaviours (body mass index (BMI), smoking, physical activity and diet). Cardiovascular disease remains the leading cause of death worldwide [4] , and the majority of the *Life's Simple 7* modifiable risk factors have been associated with longevity in previous prospective observational studies [5, 6, 7, 8, 9, 10, 11]. Yet, from these observational studies it is not possible to infer causality, due to potential reverse causation bias and residual confounding.

In Mendelian randomization (MR), genetic variants that modulate the risk factors of interest are used as instrumental variables [12]. Genetic variants are randomly allocated during meiosis and cannot be changed throughout life; thus, genetic associations are somewhat protected from reverse causation bias and residual confounding. Consequently, it is possible to address causal hypotheses using the MR design.

The MR design has been previously used to investigate causality between several cardiovascular risk factors and parental lifespan [13]. Because offspring shares 50% of its genome with each parent, parental life span can be used as a proxy outcome. A limitation of this kin‐cohort design in general is that parents have to reach their reproductive age to be included. Furthermore, parental life span in the previous MR study was self‐reported, which might have introduced misclassification bias. Recently, an individual‐level genome‐wide association study (GWAS) on longevity was published [14]. This enables the investigation of cardiovascular risk factors and lifestyle behaviours without the additional assumptions of a kin‐cohort design.

The objective of this study was to investigate causal associations between multiple potentially modifiable risk factors and longevity using the MR design. We investigated the cardiovascular risk factors and lifestyle behaviours as described in *Life's Simple 7*, as well as educational level as measure of socio‐economic status and sleep as novel lifestyle‐related risk factor.

## Methods

### Two‐sample MR design

We used a two‐sample MR design: a genetic instrumental variable analysis based on summary‐level data with single‐nucleotide polymorphisms (SNPs) as instruments for the risk factor. To obtain unbiased estimates of the causal effects, it is essential that the MR assumptions hold. These assumptions include the following: (i) the SNPs are associated with the exposure; (ii) the SNPs are independent of confounders of the risk factor–outcome association; and (iii) the SNPs influence the outcome only via the exposure [12].

### Data sources for and selection of the genetic instruments

We identified genetic instruments for each modifiable risk factor by considering the largest GWAS or meta‐analysis conducted primarily amongst individuals of European ancestry. Details on the data sources from which we obtained the instrumental variables can be found in Table 1.

**Table 1:** Overview of the data sources of the instrumental variables used in the Mendelian randomization study

Risk factor	Sample size	Ancestry	Unit^a^	SNPs^b^	Variance (%)^c^	Overlap^d^
Glucose						
Type 2 diabetes [15]	898 130	European	odds of type 2 diabetes	285/403	16.3% for 403 SNPs	~10‐20%
Fasting glucose [16]	133 010	European	mmol L^‐1^	35/36	4.8% for 36 SNPs	None
Blood pressure						
Systolic blood pressure [17]	>1 million	European	mmHg	242/362	5.66% for 362 SNPs	~20‐25%
Diastolic blood pressure [17]	>1 million	European	mmHg	300/405	5.32% for 405 SNPs	~20‐25%
Cholesterol						
LDL cholesterol [18]	188 577	primarily European	1‐SD increase in LDL cholesterol	53/58	14.6% for 58 SNPs	None
HDL cholesterol [18]	188 577	primarily European	1‐SD increase in HDL cholesterol	64/71	13.7% for 71 SNPs	None
Triglycerides [18]	188 577	primarily European	1‐SD increase in triglycerides	35/40	11.7% for 40 SNPs	None
Overweight						
Body mass index [19]	~700 000	European	1‐SD increase in body mass index	842/941	~6.0% for 941 SNPs	~30‐35%
Smoking						
Smoking initiation [20]	1 232 091	European	ever smoked regularly compared to never smoked	357/378	2.3% for 378 SNPs	~15‐25%
Cigarettes per day [20]	337 334	European	1‐SD increase in number of cigarettes smoked per day	46/55	~1% for 55 SNPs	~15‐25%
Diet						
Alcohol consumption [20]	941 80	European	1‐SD increase in log‐transformed alcoholic drinks/week	89/99	~0.2% for 99 SNPs	~15‐25%
Alcohol dependence [21]	46 568	European	odds of alcohol dependence	3/3	~0.4% for 3 SNPs	None
Coffee consumption [22]	375 833	European	50% change	14/15	~0.5% for 15 SNPs	None
Physical activity						
MVPA [23]	377 234	European	1‐SD increase in MET‐minutes/week of MVPA	5/9	0.073% for 9 SNPs	None
Sedentary behaviour [24]	91 105	European	1‐SD increase in sedentary time	4/4	0.08% for 4 SNPs	None
Sleep						
Insomnia [25]	1 331 010	European	odds of insomnia	237/248	2.6% for 248 SNPs	None
Sleep duration [26]	446 118	European	hour per day	77/78	0.69% for 78 SNPs	None
Short sleep duration [26]	411 934	European	<7 h compared to 7–8 h per day	26/27	NA	None
Long sleep duration [26]	339 926	European	≥9 h compared to 7–8 h per day	7/8	NA	None
Education						
Educational level [27]	1 131 881	European	1‐SD increase in years of educational attainment	1196/1271	11‐13% for 1271 SNPs	~15‐30%

MET, metabolic equivalent of task; MVPA, moderate‐to‐vigorous physical activity; NA, not available; SD, standard deviation; SNP, single‐nucleotide polymorphism.

^a^ Units as used in the MR analysis.

^b^ Number of SNPs included in MR/number of SNPs identified in GWAS.

^c^ The phenotypic variance explained by the genetic instruments, as reported in the risk factor GWASs.

^d^ The estimated overlap of the longevity GWAS with the risk factor GWASs. The percentages represent the part of the total number of longevity cases and controls derived from overlapping sources.

SNPs were selected as instrumental variables if associated with the modifiable risk factors at the genome‐wide significance threshold (P < 5×10^‐8^). SNPs located in or close to the *APOE* or *FOXO3* genes were excluded, as these genes are known to be strongly related to longevity via multiple pathways. If SNPs within each trait were in linkage disequilibrium (*r* ^2^ > 0.1), we included the SNP with the strongest correlation to the exposure (e.g. the smallest *P*‐value). Finally, we excluded SNPs that were not available in the longevity GWAS.

**Fig. 1:**
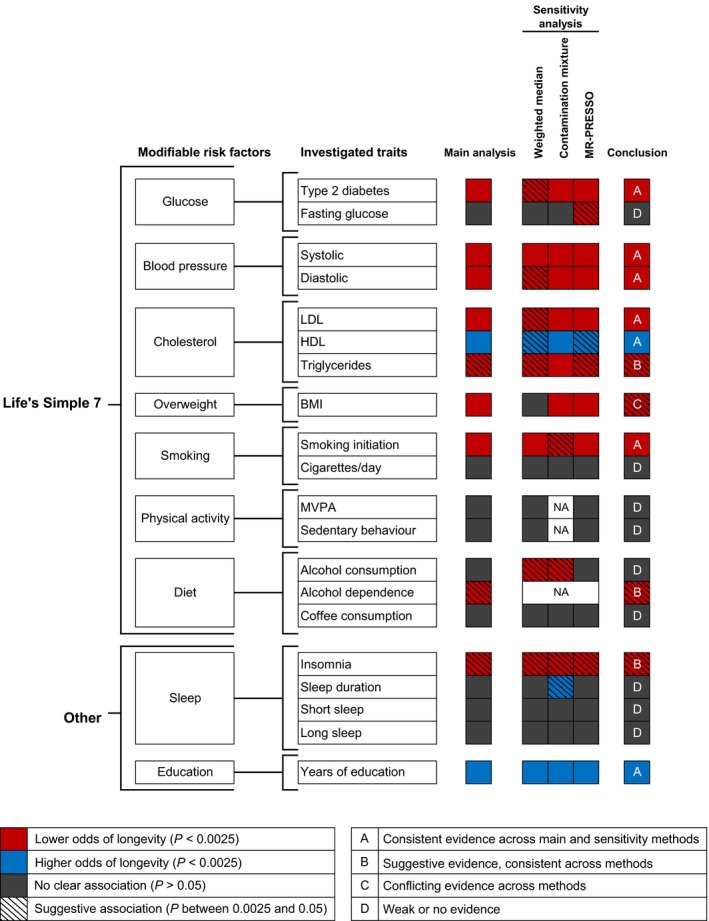
Overview of the design and results of this MR study on modifiable risk factors and longevity.*Life’s Simple 7*according to the American Heart Association’s initiative [3]. The inverse variance‐weighted method was used for the main analysis. All results can be found in Fig. 2 and Table S2. Abbreviations: BMI, body mass index; HDL, high‐density lipoprotein; LDL, low‐density lipoprotein; MVPA, moderate‐to‐vigorous physical activity; NA, not applicable due to limited number of SNPs.

An overview of the modifiable risk factors and corresponding traits included in this MR study has been provided in Fig. 1. We included SNPs for type 2 diabetes (*N* = 285) [15], fasting glucose (*N* = 35) [16], systolic blood pressure (SBP) (*N* = 242) and diastolic blood pressure (DBP) (*N* = 300) [17], lipids (low‐density lipoprotein (LDL) (*N* = 53) and high‐density lipoprotein (HDL) (*N* = 64) cholesterol and triglycerides (*N* = 35) [18]), BMI (*N* = 841) [19], smoking (initiation (*N* = 357) and heaviness (*N* = 46) [20]), alcohol consumption (*N* = 89) [20], alcohol dependence (*N* = 3) [21] coffee consumption (*N* = 14) [22], physical activity (moderate‐to‐vigorous physical activity (MVPA) (*N* = 5) [23] and sedentary behaviour (*N* = 4) [24]), insomnia (*N* = 237) [25], sleep duration (overall (N = 77) and short (*N* = 26, <7 h vs. 7–8 h) and long (*N* = 7, ≥9 h vs. 7–8 h) sleep duration) [26] and educational level (*N* = 1196) [27]. The phenotypic variance explained by the genetic instruments varied from 0.073% for MVPA to 16.3% for type 2 diabetes (Table 1).

### Data source for longevity

A recently published GWAS meta‐analysis on longevity was used to extract the genetic associations with longevity [14]. This meta‐analysis included European ancestry participants from ~20 population‐based or family‐based cohorts in the United States and Europe (i.e. Denmark, Finland, France, Iceland, Italy, the Netherlands and United Kingdom). Cases were individuals who lived beyond an age at or above the 90^th^ survival percentile (*N* = 11 262), based on cohort life tables from census data from the appropriate country, sex and birth cohort. Controls (*N* = 25 483) were individuals who died at or before the age at the 60^th^ survival percentile or whose age at the last follow‐up visit was at or before the 60^th^ survival percentile. Many of the included cohorts recruited individuals who were already relatively old at the start of follow‐up [14]. Therefore, for many studies the number of controls was small in comparison to the number of cases.

To harmonize the data from the exposure and longevity GWASs, the effect estimates of the SNPs with unmatched effects and other alleles were flipped. The present study was approved by the Swedish Ethical Review Authority.

### Statistical analyses

We used the inverse variance‐weighted (IVW) method as main analysis method. To obtain one causal IVW estimate for each exposure, Wald ratio estimates of different SNPs were combined in a multiplicative random‐effects meta‐analysis [12]. This method leads to precise causal estimates, but these might be affected by pleiotropy or invalid instrument bias in case not all MR assumptions hold. Therefore, we used several sensitivity analyses to evaluate the robustness of the results and check for pleiotropy: the weighted median method, contamination mixture method, MR‐Egger regression and Mendelian Randomization Pleiotropy RESidual Sum and Outlier (MR‐PRESSO). The weighted median method is a method to check invalid instrument bias and provides a consistent causal estimate if over 50% of the weight in the analysis is from valid SNPs [28]. The contamination mixture method is a robust method if the largest group of SNPs estimating the same quantity is the group of valid instruments [29]. MR‐Egger regression can detect and adjust for directional pleiotropy, but has low precision [28]. The MR‐PRESSO method evaluates whether exclusion of potential outlier SNPs influences the results, which is an indication of potential pleiotropy [30].

The genetic instruments for LDL cholesterol, HDL cholesterol and triglycerides are partly overlapping [18]. Therefore, we performed multivariable MR as a sensitivity analysis to obtain causal estimates adjusted for this genetic correlation.

In case a significant causal effect was observed in the main analysis, we used multivariable MR to assess whether the association was potentially mediated by the major noncommunicable diseases type 2 diabetes and cardiovascular disease (CVD) [31]. We assessed the attenuating effects after adjustment for each disease separately. For CVD, we used GWAS summary data from the FinnGen Study [32] and for type 2 diabetes from the DIAbetes Genetics Replication And Meta‐analysis (DIAGRAM) consortium [5].

We performed post hoc power calculations for the main IVW analyses using an online power calculation tool (https://sb452.shinyapps.io/power/) (Table S1) [33].

The statistical analyses were conducted in RStudio (Version 1.2.5019) with the R packages MendelianRandomization [34] and MRPRESSO [30]. Results were reported as odds ratios (OR) with corresponding 95% confidence intervals (CI). To adjust for multiple testing, we used a Bonferroni‐corrected, two‐sided significance level of 0.0025 (0.05 divided by 20 risk factors). P‐values above the Bonferroni‐corrected significance level, but below 0.05 were considered as suggestive for a potential association.

## Results

### Modifiable risk factors and longevity: main results

**Fig. 2:**
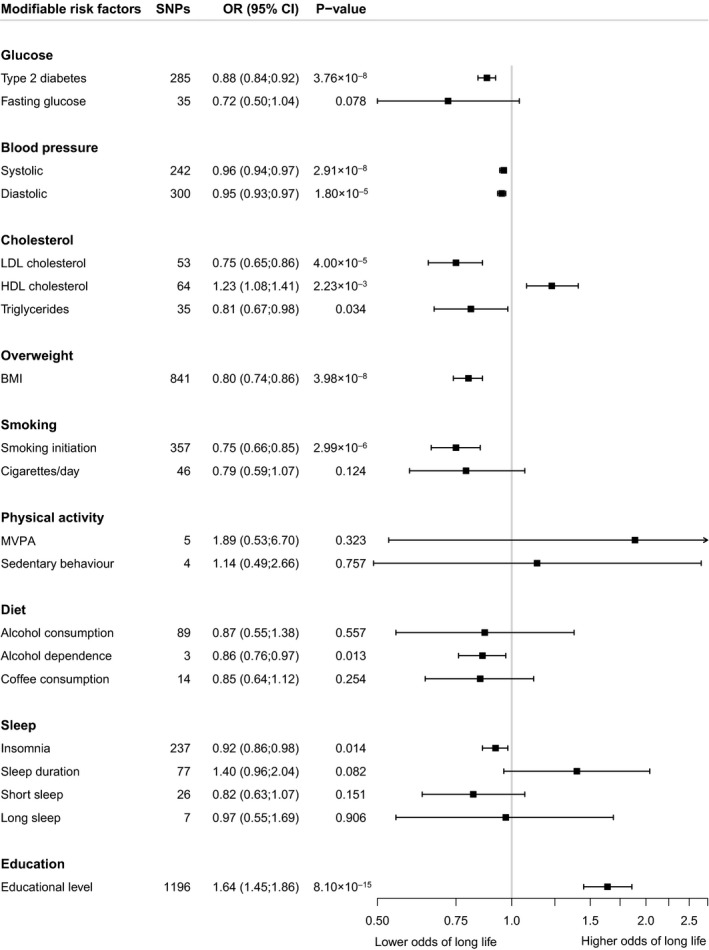
The association between modifiable risk factors and longevity beyond the 90^th^percentile using the inverse variance‐weighted Mendelian randomization method. Odds ratios represent the associations with longevity of respectively: type 2 diabetes; 1‐mmol L^‐1^increase in fasting glucose; 1‐mmHg increase in SBP; 1‐mmHg increase in DBP; 1‐SD increase in LDL cholesterol; 1‐SD increase in HDL cholesterol; 1‐SD increase in triglycerides; 1‐SD increase in BMI; ever smoked regularly compared to never smoked; 1‐SD increase in number of cigarettes smoked per day; 1‐SD increase in log‐transformed alcoholic drinks/week; alcohol dependence; 50%‐change in coffee consumption; 1‐SD increase in MET‐minutes/week of MVPA; 1‐SD increase in sedentary time; insomnia; 1‐hour/day increase in sleep duration; <7 h sleep duration compared to 7–8 h; ≥9 h sleep duration compared to 7–8 h; 1‐SD increase in years of educational attainment. Abbreviations: BMI, body mass index; HDL, high‐density lipoprotein; LDL, low‐density lipoprotein; MVPA, moderate‐to‐vigorous physical activity; OR, odds ratio.

Risk factors that were associated with a lower odds of longevity included (Fig. 2): genetic liability to type 2 diabetes (OR 0.88; CI: 0.84;0.92), genetically predicted higher systolic and diastolic blood pressure (OR per 1‐mmHg increase: 0.96; CI 0.94;0.97 for SBP and 0.95; CI: 0.93;0.97 for DBP), genetically predicted higher LDL cholesterol (OR per 1‐SD increase: 0.75; CI: 0.65;0.86), genetically predicted higher BMI (OR per 1‐SD increase: 0.80; CI: 0.74;0.86) and genetic liability to smoking initiation (OR 0.75; CI: 0.66;0.85). A higher odds of longevity was observed for genetically predicted HDL cholesterol (OR per 1‐SD increase: 1.23; CI: 1.08;1.41) and for educational level (OR per 1‐SD increase: 1.64, CI: 1.45;1.86). The following genetically predicted risk factors were suggestively associated with a lower odds of longevity: higher triglycerides (OR per 1‐SD increase: 0.81; CI 0.67;0.98), alcohol dependence (OR 0.86; CI: 0.76;0.97) and insomnia (OR 0.92; CI 0.86;0.98). No significant associations with longevity were observed for genetically predicted fasting glucose, smoking heaviness, MVPA, sedentary behaviour, alcohol consumption, coffee consumption and sleep duration (Fig. 2).

### Results sensitivity analyses to assess the MR assumptions

Potential pleiotropy was indicated by the MR‐Egger analyses of type 2 diabetes and BMI, as the intercepts significantly deviated from zero (Table S2), but the MR‐PRESSO analysis did not identify any outlying SNPs. For fasting glucose, the SNP rs6943153 was classified as an outlier in the MR‐PRESSO analysis. Exclusion of this SNP revealed a trend between higher genetically predicted fasting glucose and a lower odds of longevity (OR 0.68; CI: 0.49;0.96). For alcohol consumption, a trend towards an inverse association was revealed by the weighted median (OR 0.46; CI: 0.25;0.83) and contamination mixture method (OR 0.50; CI: 0.31;0.80). Results for the other modifiable risk factors were robust across the sensitivity analyses (Table S2).

Adjusting for the genetic correlation between the different lipids led to a partial attenuation of the association between HDL cholesterol and longevity (OR 1.15; CI: 0.98;1.34) and to a full attenuation of the suggestive association between triglycerides and longevity (OR 1.02; 95% CI: 0.81;1.29). The estimate for LDL cholesterol was marginally affected (OR 0.76; CI: 0.66;0.89).

### Mediation by major noncommunicable diseases

A simplified overview of the hypothesized relationships between the different cardiovascular risk factors, lifestyle behaviours, mediating diseases and longevity has been depicted in Fig. S1. We observed an attenuation of the association between DBP and longevity after adjustment for CVD (OR 0.97; CI: 0.95;1.00) (Fig. S2). Likewise, the association between BMI and longevity attenuated after adjustment for diabetes (OR 0.90; CI: 0.82;0.99) and partially attenuated after adjustment for CVD (OR 0.83; CI: 0.76;0.90). The effects of the other risk factors on longevity were not or only partially affected by adjustment for diabetes or CVD.

## Discussion

In this MR study, eight of the twenty investigated modifiable risk factors were significantly associated with longevity. A higher genetically predicted HDL cholesterol and educational level were associated with a higher odds of being long‐lived, whereas type 2 diabetes, SBP, DBP, LDL cholesterol, BMI and smoking initiation were associated with a lower odds of longevity. For an additional three risk factors – triglycerides, alcohol dependence and insomnia – we found suggestive evidence for a causal association with a lower odds of longevity. For the majority of the *Life's Simple 7* modifiable risk factors – except for physical activity and certain dietary factors – we found sufficient evidence for a causal association with longevity.

### Interpretation of the findings

Blood pressure, LDL cholesterol, smoking initiation and educational level were robustly associated with longevity in this MR study without indication of violation of the MR assumptions. Moreover, the associations were in similar direction as previously reported by prospective observational studies [5, 7, 8, 10, 11] and by the previous MR study on cardiovascular risk factors and parental longevity [13], although they used a smaller number of SNPs as instrumental variables. Taken together, these traditional cardiovascular risk factors are likely to be causally associated with longevity.

This MR study does not support the so‐called obesity paradox: a survival benefit in people with a higher BMI, which has been previously found in observational studies amongst people with chronic diseases [35]. However, the setting of this MR study was in a general population. The observational literature amongst the general population indicates an increased mortality risk in overweight and obese individuals [36] and is thus in line with our findings of a harmful effect of higher BMI on longevity. Moreover, a previous MR study found a linear dose–response relation of BMI with mortality in never smokers and a J‐shaped relation in ever smokers [37]. This strengthens the evidence that the obesity paradox in previous observational studies is a product of bias by confounding through smoking and reverse causation [38]. The effect of BMI is most likely partially driven by vertical pleiotropic effects via type 2 diabetes, since potential pleiotropy was indicated by the MR‐Egger regression and the association between BMI and longevity attenuated after adjustment for the genetic correlation with type 2 diabetes. Moreover, hypertension and CVD could also play a role. Nevertheless, if overweight reduction provides a longevity benefit via effects on diabetes or CVD, this should still be advocated as public health goal.

The suggestively harmful effect of higher triglycerides on longevity we observed in our study can be explained by the overlap in the genetic instruments of the different lipids. As the causal estimate for HDL also attenuated after adjustment for this genetic overlap, this suggests that mainly high LDL cholesterol affects longevity.

Our observation that alcohol dependence, based on SNPs situated in the alcohol dehydrogenase region, was suggestively associated with a lower odds to be long‐lived, whilst no clear association was observed between alcohol consumption and longevity, might suggest that especially excessive drinking affects longevity. However, we cannot rule out that the U‐shaped association between alcohol and mortality according to the observational literature [39] has been the result of residual confounding or reverse causation, as our MR study was not designed to reveal U‐shaped associations.

Drawing causal conclusions on the suggestively harmful association between insomnia and longevity observed in this MR study is not yet possible. A recent meta‐analysis of observational studies on insomnia and mortality has been inconclusive (pooled HR 1.07; CI: 0.96;1.19) with high heterogeneity between studies [40]. For triangulation, it is necessary that future observational cohorts and future MR studies reduce heterogeneity by improving the definition and assessment of insomnia, and that future MR studies further improve precision by acquiring larger sample sizes for either insomnia or longevity.

We are also not able to draw conclusions on causality yet on the association of smoking heaviness, coffee consumption, physical activity and sleep duration with longevity. Although these risk factors have been associated with longevity or mortality in observational studies [5, 7, 8, 9, 41, 42], it remains unclear whether the null associations observed in our MR study were due to the absence of a causal effect or related to the low power of these analyses (7–38%) (Table S1) because of the small phenotypic variance explained by the genetic instruments (varying from 0.073% to ~1%) (Table 1). The latter is supported by the wide confidence intervals for the causal estimates of these risk factors across all analyses.

### Underlying mechanisms

Cardiovascular diseases and type 2 diabetes are probably on many of the pathways from risk factor to longevity, as was also observed in our mediation analyses. Modification of the cardiovascular risk factors and lifestyle behaviours potentially reduces or delays the onset of noncommunicable diseases, which in turn benefits longevity. Yet, a part of the associations could not be explained by the onset of diabetes and cardiovascular disease. This might suggest that other noncommunicable diseases play a role as well, such as chronic respiratory diseases, certain types of cancer, psychiatric illnesses or Alzheimer's disease. Another explanation might be that modifiable risk factors not only influence disease onset, but also influence disease progression or disease severity, which was not captured in our mediation analyses. Finally, it might also implicate that there is a potential disease‐independent effect of these risk factors on longevity, for example via influences on other determinants of health, such as functional status, cognition or frailty [43].

The modifiable risk factor education is a more upstream determinant of health. It is likely that several pathways play a role in the beneficial effect of high educational level on longevity. Higher education might lead to more knowledge and skills to make healthier and more long‐term choices regarding lifestyle and prevention of diseases. This is reflected by a previous MR study showing that a large part of the association between education and cardiovascular disease can be explained by BMI and smoking [44]. Other contributing factors might include more resources to maintain a healthy lifestyle, less exposure to occupation‐related health hazards and better access to health care.

### Implications

The *Life's Simple 7* modifiable risk factors glucose, blood pressure, cholesterol, overweight and smoking are causally related to longevity. Modifying these risk factors can potentially improve health: partly via effects on cardiovascular diseases and type 2 diabetes, but our study also implicates an independent effect of these risk factors on longevity. As each risk factor has its own causal effect on longevity, all risk factors are potential prevention targets. This study provides evidence for governmental policy makers to improve public health by implementing prevention strategies as the *Life's Simple 7* and to reduce education inequalities in the population.

Larger sample sizes for either the exposure or outcome GWAS are needed to be able to draw causal conclusions on certain cardiovascular risk factors, but mostly lifestyle behaviours such as physical activity and alcohol consumption. Longer follow‐up durations of cohorts with genetic data are needed for acquiring larger sample sizes on the individual longevity phenotype. Our findings were similar to those of the parental life span MR [13], which implicates that using a parental longevity phenotype would be an effective strategy for increasing sample sizes in the nearby future.

### Strengths and limitations

The validity of causal inference from this MR study largely depends on whether the instrumental variable assumptions hold. The majority of our results were robust to a wide variety of sensitivity analyses to assess potential invalid instrument bias and pleiotropy. Moreover, we were able to include multiple, independent, genome‐wide significant SNPs as instruments for the different risk factors to ensure that the first MR assumption was fulfilled. Lastly, the longevity phenotype used as outcome in this study was derived from a homogeneous case–control GWAS meta‐analysis due to standardization of the survival percentiles according to sex, country and birth cohort.

It is important to acknowledge that the small variation in exposure explained by the genetic instruments for smoking heaviness, alcohol and coffee consumption, physical activity, and sleep duration resulted in low precision. Consequently, these null associations cannot be interpreted as no causal effects. Another shortcoming is that participants had to survive to a certain age to be included in many of the cohorts from the longevity GWAS meta‐analysis. This potentially led to an underrepresentation of people with early mortality in the control group and thus to an underestimation of the true effect if those people had more unfavourable risk factors. Moreover, there was a maximum of ~30–35% overlap between the GWAS for longevity and the GWASs for eight of the twenty risk factors (Table 1). Although the overlap was only partial, this might have inflated the type 1 error rate. Another limitation is that we were only able to evaluate alcohol and coffee consumption as dietary factors, because no robust and specific genetic instruments are currently available for other dietary components. Finally, our findings are only generalizable to populations of European ancestry.

## Conclusions

This MR study provided evidence that most of the *Life's Simple 7* modifiable risk factors and educational level are causally related to longevity. Part of these effects are driven by mainly cardiovascular diseases and type 2 diabetes, but there is an independent effect of the risk factors on longevity as well. Prevention strategies should focus on modifying these risk factors and reducing education inequalities to improve cardiovascular health and longevity.

## Author contribution

Sabine van Oort: Conceptualization (equal); Formal analysis (equal); Funding acquisition (lead); Investigation (equal); Methodology (supporting); Visualization (equal); Writing‐original draft (lead). Joline WJ Beulens: Conceptualization (equal); Funding acquisition (supporting); Investigation (equal); Supervision (equal); Visualization (supporting); Writing‐review & editing (equal). Adriana J. van Ballegooijen: Conceptualization (supporting); Funding acquisition (supporting); Investigation (equal); Supervision (supporting); Validation (supporting); Writing‐review & editing (equal). Stephen Burgess: Investigation (equal); Methodology (equal); Software (lead); Supervision (supporting); Visualization (equal); Writing‐review & editing (equal). Susanna Larsson: Conceptualization (equal); Formal analysis (equal); Funding acquisition (lead); Investigation (equal); Methodology (lead); Supervision (lead); Visualization (equal); Writing‐review & editing (equal).

## Funding

This work was supported by the Amsterdam Public Health Research Institute [two personal travel grants to S.O.], the Swedish Research Council [Vetenskapsrådet; 2019‐00977 to S.C.L.], and the Wellcome Trust and the Royal Society [204623/Z/16/Z to S.B.]. This work was supported by funding from the National Institute for Health Research [Cambridge Biomedical Research Centre at the Cambridge University Hospitals National Health Service Foundation Trust]. The views expressed are those of the authors and not necessarily those of the National Health Service, the National Institute for Health Research or the Department of Health and Social Care. The funding bodies had no role in the design of the study, the collection, analysis and interpretation of data, or in writing the manuscript.

## Competing interests

The authors declare that they have no competing interests.

## Supporting information


**Figure S1.** Simplified overview of the hypothesized associations of modifiable cardiovascular and lifestyle risk factors with longevity.
**Figure S2.** Mediation of the association between modifiable risk factors and longevity by diabetes and cardiovascular disease.
**Table S1.** Post‐hoc power calculation for the IVW analyses on modifiable risk factors and longevity.
**Table S2.** Sensitivity analyses of the Mendelian randomization study on modifiable risk factors and longevity.

## Data Availability

All data analysed in this study are based on publicly available summary statistics data provided by genetic consortia.
