# Supplementary material for: Cardiovascular risk factors and lifestyle behaviours in relation to longevity: a Mendelian randomization study
Source: J Intern Med. 2020 Nov 13;289(2):232–43. doi: 10.1111/joim.13196 (PMC7894570; doi:10.1111/joim.13196)
Supplement: Supplementary file 1 — Figure S1. Simplified overview of the hypothesized associations of modifiable cardiovascular and lifestyle risk factors with longevity. Figure S2. Mediation of the association between modifiable risk factors and longevity by diabetes and cardiovascular disease. Table S1. Post‐hoc power calculation for the IVW analyses on modifiable risk factors and longevity. Table S2. Sensitivity analyses of the Mendelian randomization study on modifiable risk factors and longevity. [file JOIM-289-232-s001.docx]

**Supplementary Materials**

**Cardiovascular risk factors and lifestyle behaviours in relation to longevity: a Mendelian randomization study**

Sabine van Oort, MD, Joline W.J. Beulens, PhD, Adriana J. van Ballegooijen, PhD,

Stephen Burgess, PhD, Susanna C. Larsson, PhD

|  | Page |
| --- | --- |
| **Supplementary Table 1.** Post-hoc power calculation for the IVW analyses on modifiable risk factors and longevity. | 2 |
| **Supplementary Table 2.** Sensitivity analyses of the Mendelian randomization study on modifiable risk factors and longevity. | 4 |
| **Supplementary Figure 1.** Simplified overview of the hypothesized associations of modifiable cardiovascular and lifestyle risk factors with longevity. | 6 |
| **Supplementary Figure 2.** Mediation of the association between modifiable risk factors and longevity by diabetes and cardiovascular disease. | 7 |
| **Supplementary references** | 8 |

**Supplementary Table 1. Post-hoc power calculation for the IVW analyses on modifiable risk factors and longevity.**

| **Modifiable risk factor** | **Sample size*** | **Ratio cases to controls*** | **R^2^ of exposure by genetic variants**† | **Causal effect**‡  **(OR according to IVW)** | **Significance level** | **Power§** |
| --- | --- | --- | --- | --- | --- | --- |
| **Glucose** |  |  |  |  |  |  |
| Type 2 diabetes | 36,745 | 1:2.3 | 0.163 | 0.88 | 0.0025 | **94%** |
| Fasting glucose | 36,745 | 1:2.3 | 0.048 | 0.84^a^ | 0.0025 | **63%** |
| **Blood pressure** |  |  |  |  |  |  |
| Systolic blood pressure | 36,745 | 1:2.3 | 0.0566 | 0.43^b^ | 0.0025 | **100%** |
| Diastolic blood pressure | 36,745 | 1:2.3 | 0.0532 | 0.56^c^ | 0.0025 | **100%** |
| **Cholesterol** |  |  |  |  |  |  |
| LDL cholesterol | 36,745 | 1:2.3 | 0.0146 | 0.75 | 0.0025 | **52%** |
| HDL cholesterol | 36,745 | 1:2.3 | 0.0137 | 1.23 | 0.0025 | **19%** |
| Triglycerides | 36,745 | 1:2.3 | 0.0117 | 0.81 | 0.0025 | **16%** |
| **Overweight** |  |  |  |  |  |  |
| Body mass index | 36,745 | 1:2.3 | 0.06 | 0.80 | 0.0025 | **96%** |
| **Smoking** |  |  |  |  |  |  |
| Smoking initiation | 36,745 | 1:2.3 | 0.023 | 0.75 | 0.0025 | **79%** |
| Cigarettes per day | 36,745 | 1:2.3 | 0.01 | 0.79 | 0.0025 | **17%** |
| **Physical activity** |  |  |  |  |  |  |
| MVPA | 36,745 | 1:2.3 | 0.00073 | 1.89 | 0.0025 | **7%** |
| Sedentary behaviour | 36,745 | 1:2.3 | 0.0008 | 1.14 | 0.0025 | **0.3%** |
| **Diet** |  |  |  |  |  |  |
| Alcohol consumption | 36,745 | 1:2.3 | 0.002 | 0.87 | 0.0025 | **0.7%** |
| Alcohol dependence | 36,745 | 1:2.3 | 0.004 | 0.86 | 0.0025 | **0.5%** |
| Coffee consumption | 36,745 | 1:2.3 | 0.0048 | 0.85^d^ | 0.0025 | **NA** |
| **Sleep duration** |  |  |  |  |  |  |
| Insomnia | 36,745 | 1:2.3 | 0.026 | 0.92 | 0.0025 | **3%** |
| Sleep duration | 36,745 | 1:2.3 | 0.0069 | 1.45^e^ | 0.0025 | **38%** |
| Short sleep duration | 36,745 | 1:2.3 | NA | 0.82 | 0.0025 | **NA** |
| Long sleep duration | 36,745 | 1:2.3 | NA | 0.97 | 0.0025 | **NA** |
| **Education** |  |  |  |  |  |  |
| Educational level | 36,745 | 1:2.3 | 0.11 | 1.64 | 0.0025 | **100%** |
|  |  |  |  |  |  |  |

* Sample size and ratio cases to controls according to the outcome GWAS on longevity

† Variance explained of exposure by genetic variants as reported in GWASs on the different modifiable risk factors

‡ OR per 1-SD change for continuous exposures

§ Calculated using an online power calculation tool (<https://sb452.shinyapps.io/power/>) [1]

^a^ OR per 1-SD increase in fasting glucose, using an SD of 0.5 mmol/L approximated from the study characteristics in the GWAS meta-analysis [2]

^b^ OR per 1-SD increase in SBP, using an SD of 20.7 mmHg as reported in the UK Biobank part of the GWAS meta-analysis [3]

^c^ OR per 1-SD increase in DBP, using an SD of 11.3 mmHg as reported in the UK Biobank part of the GWAS meta-analysis [3]

^d^ OR per 50% change in coffee consumption. SD was not provided in GWAS meta-analysis [4]

^e^ OR per 1-SD increase in sleep duration, using an SD of 1.1 h as reported in the GWAS [5]

Abbreviations: BMI = body mass index; IVW = inverse-variance weighted; LDL = low-density lipoprotein; HDL = high-density lipoprotein; MVPA = moderate-to-vigorous physical activity; OR = odds ratio.

**Supplementary Table 2. Sensitivity analyses of the Mendelian randomization study on modifiable risk factors and longevity.**

|  |  | **Sensitivity analyses** | | | | | | | | | | |
| --- | --- | --- | --- | --- | --- | --- | --- | --- | --- | --- | --- | --- |
|  |  | **Weighted median** | | **Contamination mixture** | | **MR-Egger** | | | | **MR-PRESSO** | | |
| **Risk factors** | **SNPs** | OR (95% CI) | P-value | OR (95% CI) | P-value | OR (95% CI) | P-value | Intercept (95% CI) | | SNPs | OR (95% CI) | P-value |
| **Glucose** |  |  |  |  |  |  |  |  | |  |  |  |
| Type 2 diabetes | 285 | 0.90 (0.83;0.98) | ***1.33E-2*** | 0.84 (0.78;0.90) | **1.68E-6** | 1.00 (0.90;1.10) | 0.971 | -0.009 (-0.015;-0.003) | | 285 | 0.88 (0.84;0.92) | **3.76E-8** |
| Fasting glucose | 35 | 0.76 (0.49;1.18) | 0.221 | 0.53 (0.34;1.25) | 0.128 | 0.88 (0.45;1.72) | 0.703 | -0.007 (-0.025;0.012) | | 34* | 0.68 (0.49;0.96) | ***0.0359*** |
| **Blood pressure** |  |  |  |  |  |  |  |  | |  |  |  |
| Systolic blood pressure | 242 | 0.96 (0.94;0.98) | **8.39E-4** | 0.94 (0.91;0.96) | **8.38E-5** | 0.95 (0.89;1.01) | 0.095 | 0.002 (-0.011;0.014) | | 242 | 0.96 (0.94;0.97) | **2.91E-8** |
| Diastolic blood pressure | 300 | 0.95 (0.92;0.98) | ***0.001*** | 0.91 (0.87;0.95) | **9.54E-5** | 1.02 (0.94;1.12) | 0.581 | -0.010 (-0.021;0.001) | | 300 | 0.95 (0.93;0.97) | **1.80E-5** |
| **Cholesterol** |  |  |  |  |  |  |  |  | |  |  |  |
| LDL cholesterol | 53 | 0.80 (0.67;0.95) | ***0.0106*** | 0.77 (0.68;0.86) | **1.07E-3** | 0.79 (0.62;1.02) | 0.067 | 0.003 (-0.008;0.014) | | 53 | 0.75 (0.65;0.86) | **4.00E-5** |
| HDL cholesterol | 64 | 1.22 (1.04;1.42) | ***0.013*** | 1.32 (1.15;1.70) | **6.39E-5** | 1.18 (0.96;1.45) | 0.109 | -0.004 (-0.019;0.011) | | 63* | 1.21 (1.06;1.38) | ***5.23E-3*** |
| Triglycerides | 35 | 0.78 (0.65;0.94) | ***7.72E-3*** | 0.73 (0.63;0.85) | **2.62E-4** | 0.81 (0.61;1.08) | 0.157 | 0.000 (-0.016;0.017) | | 33* | 0.82 (0.69;0.99) | ***0.0432*** |
| **Overweight** |  |  |  |  |  |  |  |  | |  |  |  |
| Body mass index | 841 | 0.89 (0.77;1.02) | 0.093 | 0.79 (0.72;0.89) | **8.91E-5** | 0.94 (0.81;1.08) | 0.357 | -0.004 (-0.007;-0.001) | | 841 | 0.78 (0.74;0.86) | **3.98E-8** |
| **Smoking** |  |  |  |  |  |  |  |  |  |  |  |  |
| Smoking initiation | 357 | 0.72 (0.60;0.86) | **2.97E-4** | 0.68 (0.54;0.91) | ***5.27E-3*** | 0.70 (0.41;1.17) | 0.171 | 0.001 (-0.008;0.011) | | 357 | 0.75 (0.66;0.85) | **2.99E-6** |
| Cigarettes per day | 46 | 0.87 (0.58;1.30) | 0.489 | 0.77 (0.51;1.14) | 0.126 | 1.24 (0.74;2.05) | 0.413 | -0.014 (-0.027;-0.001) | | 46 | 0.79 (0.59;1.07) | 0.124 |
| **Physical activity** |  |  |  |  |  |  |  |  | |  |  |  |
| MVPA | 5 | 2.19 (0.46;10.38) | 0.322 | NA | NA | 1.29E+3  (1.39E-9;1.19E15) | 0.611 | -0.092 (-0.479;0.295) | | 5 | 1.89 (0.53;6.70) | 0.323 |
| Sedentary behaviour | 4 | 1.03 (0.45;2.39) | 0.938 | NA | NA | 4.02E-5  (1.73E-13;9345) | 0.303 | 0.294 (-0.258;0.847) | | 4 | 1.14 (0.49;2.66) | 0.757 |
| **Diet** |  |  |  |  |  |  |  |  | |  |  |  |
| Alcohol consumption | 89 | 0.46 (0.25;0.83) | ***0.011*** | 0.50 (0.31;0.80) | ***0.014*** | 0.54 (0.24;1.22) | 0.137 | 0.008 (-0.003;0.019) | | 87* | 0.87 (0.57;1.32) | 0.506 |
| Alcohol dependence | 3 | NA | NA | NA | NA | NA | NA | NA | | NA | NA | NA |
| Coffee consumption | 14 | 0.89 (0.61;1.29) | 0.533 | 0.80 (0.47;1.10) | 0.162 | 0.89 (0.51;1.55) | 0.683 | -0.002 (-0.028;0.023) | | 14 | 0.85 (0.64;1.12) | 0.254 |
| **Sleep duration** |  |  |  |  |  |  |  |  | |  |  |  |
| Insomnia | 237 | 0.90 (0.81;1.00) | ***0.040*** | 0.84 (0.75;0.94) | ***4.56E-03*** | 0.81 (0.61;1.06) | 0.128 | 0.006 (-0.006;0.018) | | 237 | 0.92 (0.86;0.98) | ***0.014*** |
| Sleep duration | 77 | 1.57 (0.95;2.59) | 0.077 | 1.99 (1.13;3.59) | ***0.0225*** | 3.27 (0.78;13.74) | 0.105 | -0.014 (-0.038;0.009) | | 75* | 1.40 (1.00;1.96) | 0.051 |
| Short sleep duration | 26 | 0.83 (0.58;1.21) | 0.337 | 1.05 (0.42;1.72) | 0.738 | 0.43 (0.15;1.22) | 0.112 | 0.023 (-0.013;0.058) | | 26 | 0.82 (0.63;1.07) | 0.151 |
| Long sleep duration | 7 | 1.13 (0.71;1.78) | 0.614 | 1.55 (0.36;3.00) | 0.170 | 0.31 (0.07;1.34) | 0.116 | 0.078 (-0.016;0.173) | | 6^*^ | 1.15 (0.74;1.80) | 0.562 |
| **Education** |  |  |  |  |  |  |  |  | |  |  |  |
| Educational level | 1196 | 1.75 (1.46;2.09) | **1.09E-9** | 2.79 (2.06;3.54) | **9.68E-12** | 1.89 (1.19;2.98) | **6.46E-3** | -0.002 (-0.007;0.003) | | 1196 | 1.64 (1.45;1.86) | **8.10E-15** |

* One or more SNPs excluded.

P-values below the Bonferroni-corrected threshold of 0.0025 are displayed in bold and suggestive P-values between 0.05 and 0.0025 are displayed in bold-italic.

Odds ratios represent the associations with longevity of respectively: type 2 diabetes; 1-mmol/L increase in fasting glucose; 1-mmHg increase in SBP; 1-mmHg increase in DBP; 1-SD increase in LDL cholesterol; 1-SD increase in HDL cholesterol; 1-SD increase in triglycerides; 1-SD increase in BMI; ever smoked regularly compared to never smoked; 1-SD increase in number of cigarettes smoked per day; 1-SD increase in log-transformed alcoholic drinks/week; alcohol dependence; 50%-change in coffee consumption; 1-SD increase in MET-minutes/week of MVPA; 1-SD increase in sedentary time; insomnia; 1-hour/day increase in sleep duration; <7 hours sleep duration compared to 7-8 hours; ≥9 hours sleep duration compared to 7-8 hours; 1-SD increase in years of educational attainment.

Abbreviations: BMI = body mass index; LDL = low-density lipoprotein; HDL = high-density lipoprotein; MVPA = moderate-to-vigorous physical activity.

**
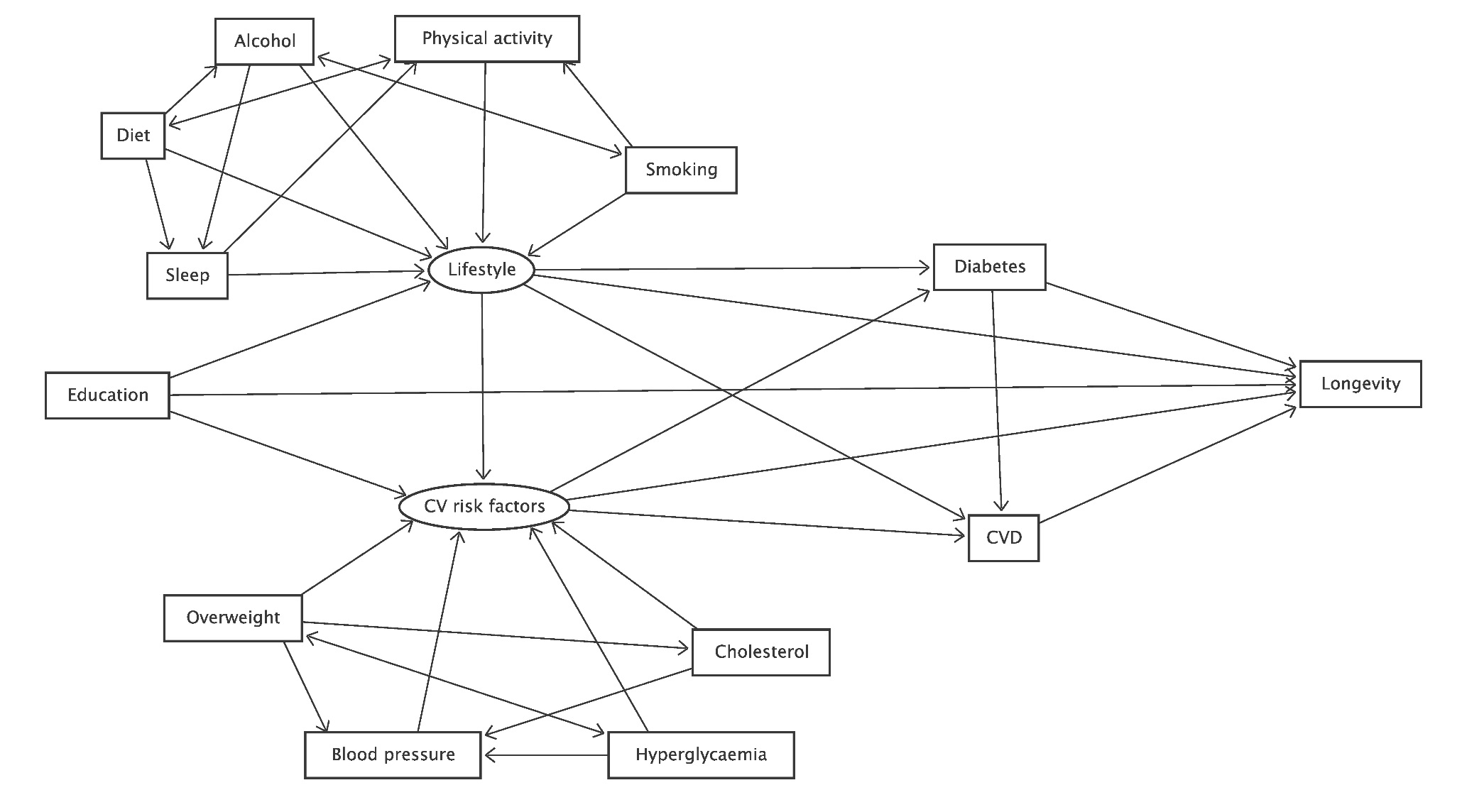
**

**Supplementary Figure 1. Simplified overview of the hypothesized associations of modifiable cardiovascular and lifestyle risk factors with longevity.**

Not all vectors between the various lifestyle behaviours and cardiovascular risk factors have been drawn in this overview for clarification purposes.

Abbreviations: CVD = cardiovascular disease; CV risk factors = cardiovascular risk factors


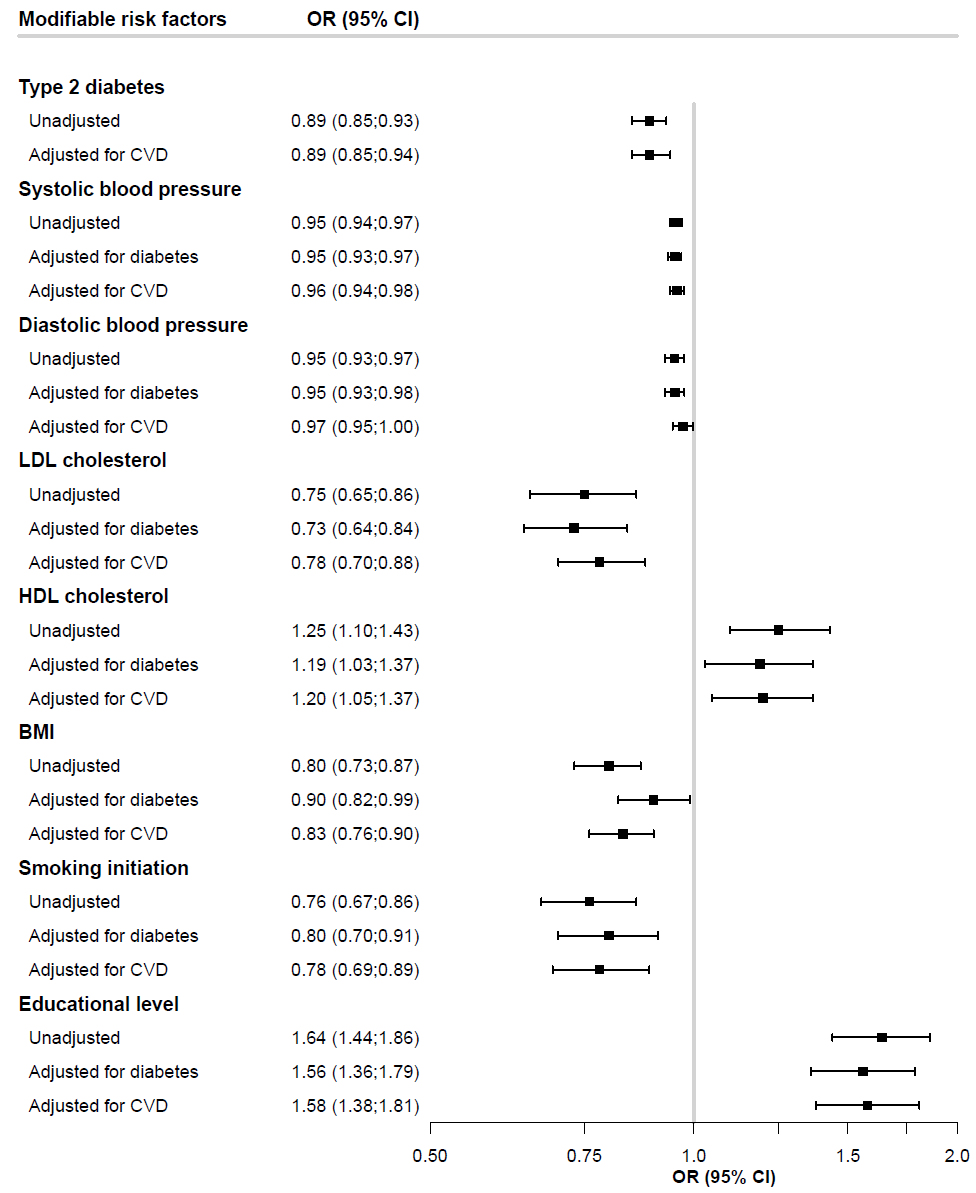
**Supplementary Figure 2. Mediation of the association between modifiable risk factors and longevity by diabetes and cardiovascular disease.**

Odds ratios represent the associations with longevity of respectively: type 2 diabetes; 1-SD increase in LDL cholesterol; 1-SD increase in HDL cholesterol; 1-SD increase in BMI; ever smoked regularly compared to never smoked; 1-SD increase in years of educational attainment.

The estimates for the unadjusted analysis slightly differ from the results presented in Figure 2 due to a different number of SNPs available in the mediator GWASs.

Abbreviations: BMI = body mass index; CI = confidence interval; CVD = cardiovascular disease; GWAS = genome-wide association study; LDL = low-density lipoprotein; N/A = not applicable; OR = odds ratio.

**Supplementary references**

1 S. Burgess. Sample size and power calculations in Mendelian randomization with a single instrumental variable and a binary outcome. *International journal of epidemiology* 2014; **43:** 922-9.

2 R. A. Scott, V. Lagou, R. P. Welch*, et al.* Large-scale association analyses identify new loci influencing glycemic traits and provide insight into the underlying biological pathways. *Nat Genet* 2012; **44:** 991-1005.

3 E. Evangelou, H. R. Warren, D. Mosen-Ansorena*, et al.* Genetic analysis of over 1 million people identifies 535 new loci associated with blood pressure traits. *Nat Genet* 2018; **50:** 1412-25.

4 V. W. Zhong, A. Kuang, R. D. Danning*, et al.* A genome-wide association study of bitter and sweet beverage consumption. *Human Molecular Genetics* 2019; **28:** 2449-57.

5 J. J. Lee, R. Wedow, A. Okbay*, et al.* Gene discovery and polygenic prediction from a genome-wide association study of educational attainment in 1.1 million individuals. *Nat Genet* 2018; **50:** 1112-21.
